# Machine learning regression algorithms to predict emissions from steam boilers

**DOI:** 10.1016/j.heliyon.2024.e26892

**Published:** 2024-02-22

**Authors:** Bárbara D. Ross-Veitía, Dayana Palma-Ramírez, Ramón Arias-Gilart, Rebeca E. Conde-García, Alejandro Espinel-Hernández, José R. Nuñez-Alvarez, Hernan Hernández-Herrera, Yolanda E. Llosas-Albuerne

**Affiliations:** a National Center for Applied Electromagnetism (CNEA), Universidad de Oriente, Ave. de Las Américas s/n, 90100, Santiago de Cuba, Cuba; b Energy Department, Universidad de la Costa, (CUC), Calle 58 # 55-66, Barranquilla, 080002, Colombia; c Faculty of Engineering, Universidad Simón Bolívar, Carrera 59 #59-132, Barranquilla, 080002, Colombia; d Electrical Engineering Department, Universidad Técnica de Manabí (UTM), Portoviejo, Manabí, 130105, Ecuador

**Keywords:** Linear regression, Python, Artificial intelligence, Combustion

## Abstract

Currently, the modeling of complex chemical-physical processes is drastically influencing industrial development. Therefore, the analysis and study of the combustion process of the boilers using machine learning (ML) techniques are vital to increase the efficiency with which this equipment operates and reduce the pollution load they contribute to the environment. This work aims to predict the emissions of CO, CO_2_, NOx, and the temperature of the exhaust gases of industrial boilers from real data. Different ML algorithms for regression analysis are discussed. The following are input variables: ambient temperature, working pressure, steam production, and the type of fuel used in around 20 industrial boilers. Each boiler's emission data was collected using a TESTO 350 Combustion Gas Analyzer. The modeling, with a machine learning approach using the Gradient Boosting Regression algorithm, showed better performance in the predictions made on the test data, outperforming all other models studied. It was achieved with predicted values showing a mean absolute error of 0.51 and a coefficient of determination of 99.80%. Different regression models (DNN, MLR, RFR, GBR) were compared to select the most optimal. Compared to models based on Linear Regression, the DNN model has better prediction performance. The proposed model provides a new method to predict CO_2_, CO, NOx emissions, and exhaust gas outlet temperature.

## 1 Introduction

Combustion processes are the main source of energy and environmental pollution. These processes are widely used in industry, electricity generation, heat production, waste management, and many other fields [1]. The industrial equipment that takes advantage of the chemical energy of the fuels and converts it into thermal energy through the combustion process is the internal combustion engines, turbines, and boilers, among others.

Boilers are energy exchange systems in which the heat generated by burning fuel is used and transferred to the water. The resulting hot water or steam flow is used in industrial processes for different purposes: heating and electric power generation using turbines, among others [2]. There are two types of boilers in terms of the distribution of the heat exchange process: pyro tubular boilers, where the hot flow (gases) goes through the tubes and the water goes through the casing, and water tube boilers, where the water goes through the tubes and the flow of hot gases goes through the casing [3].

Boilers can use different types of fuels; the most used are fossil fuels, such as coal, petroleum derivatives, and natural gas [4]. There are also boilers fed with industrial waste or biomass, which perform less than fossil fuels [5]. Nevertheless, this equipment is vital in the industrial sector and represents a fundamental part of world energy consumption.

For several years, the analysis and study of the combustion process in this type of equipment have been of great importance to increase the efficiency with which they operate and reduce the pollutant load they contribute to the environment. Despite this process's many applications and many investigations on this topic, the boiler combustion process still has many knowledge gaps. Combustion is an oxidation reaction in which fluid dynamics, chemical kinetics, heat, and mass transfer, among other unit operations, are applied [1]. For that reason, novel, modern, and alternative methods are required to research these types of processes in depth.

Manufacturers and combustion engineers generally want to know a boiler's efficiency and gas emissions for different ranges and operating conditions. To achieve this, it is necessary to carry out an extensive experimental study or a detailed modeling of the operation of this type of system [6]. However, testing boilers under all operating conditions and fuel types is virtually impossible, time-consuming, and expensive. Furthermore, it is difficult to develop an accurate model for the operation and dynamics of combustion in a boiler due to the complex variables involved in these processes [7].

Currently, modeling industrial processes through intelligent manufacturing has important results. The fourth industrial revolution, led by emerging technologies, particularly artificial intelligence (AI), is dramatically influencing industrial development. In this sense, AI is an alternative to evaluating boilers' performance and exhaust emissions. This modeling technique can estimate the desired output parameters when sufficient experimental data is provided [8].

Machine Learning (ML) is a subfield of AI and uses algorithms that allow machines to learn from a vast set of data. Classification and regression methods are within a branch of ML known as supervised machine learning and have been applied in many research fields [9].

Supervised machine learning is subject to building models from labeled data. That is data for which the target response is already known to identify patterns by relating them to the target, given certain input variables. This technique has been used in several combustion-related studies, yielding good results, especially in the last ten years [1].

Few ML studies simultaneously analyze these systems' CO, CO_2_, NO_X_, and temperature emissions as output variables [1]. That is why this work aims to model or predict the emissions of CO, CO_2_, NOx, and the temperature of the exhaust gases of industrial boilers from real data. In this study, different ML algorithms for regression analysis will be analyzed. The following are input variables: ambient temperature, working pressure, steam production, and the type of fuel used in around 20 industrial boilers.

Most ML research in combustion processes has been carried out in furnaces and for studying flames [[10], [11], [12]]. In boilers, there are a few types of research where different ML techniques are applied; most of them focus on one or two gaseous components of the exhaust emissions, mainly NOx [[13], [14], [15]]. No research reports have been found that simultaneously predict CO, CO_2_, and NOx emissions and exhaust gas temperature. Also, research on this topic is mostly based on gas emission results from a single boiler, over which the input parameters vary. No publication reports have been found in which data obtained from different boilers with similar characteristics are used for prediction. The novelty of the presented work is related to these aspects: multi-gas prediction of boiler emissions and data obtained from several boilers with similar characteristics using ML techniques.

This research aims to propose and evaluate machine learning regression algorithms to predict CO, CO_2_, NOx emissions, and gas temperature from different steam boilers with similar characteristics.

## 2 Materials and methods

This research modeled exhaust gas emissions using real data from more than 20 industrial fire-tube boilers. The independent or input variables of the model were ambient temperature, working pressure, steam production, and fuel type. The dependent or output variables were gas emissions (CO, CO_2,_ and NO_X_) and the gas outlet temperature.

These variables were selected based on their importance from the environmental point of view and combustion efficiency. For its modeling, Linear Regression (LR), Gradient Boosting (GB), Random Forest algorithms (RFA), and a deep neural model (DNN) were used to select the appropriate model to predict CO, CO_2_, NO_X_ emissions, and gas temperature.

### 2.1 Industrial data collection

For data collection, 20 industrial steam boilers were used. These boilers are in industries, hospitals, and companies in Santiago de Cuba, Cuba. For the selection of each of this equipment, the following conditions were considered.

• Fire tube boilers
• Working pressure in a range from 6 kg/cm^2^ to 13 kg/cm^2^
• Steam generation capacity from 1277 kg/h to 2500 kg/h
• Diesel fuel or fuel oil

Each boiler's emission data was collected using a Combustion Gas Analyzer TESTO 350 [16,17]. This equipment measured the emissions of CO_2_, CO, NOx, and the outlet temperature of the exhaust gases from the industrial boilers under study. The principal parameters of the gas analyzer are illustrated in Table 1.

**Table 1:** Testo 350 Combustion Gas Analyzer information [18].

Parameters	Range	Resolution	Uncertainty
CO	0 a 10000 ppm	±1 ppm	2.38
NO_x_	0 a 4000 ppm	±1 ppm	4.31
CO_2_	0 a 50% Vol.	±0,01% Vol.	0.04
TEG	0 a 10000 °C	±1 °C	0.68

**Fig. 1:**
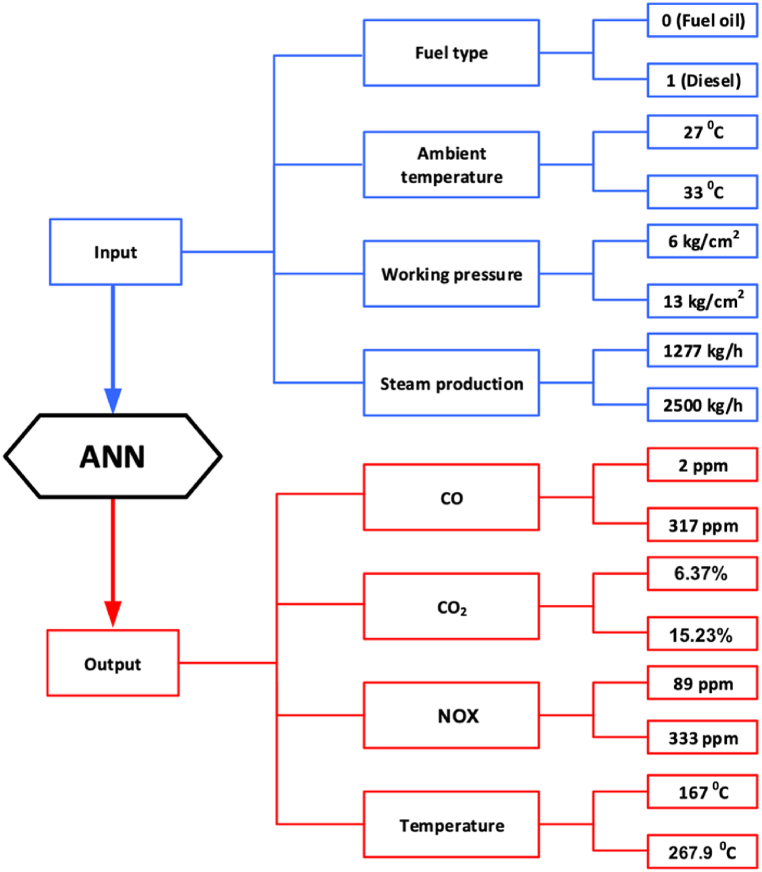
Upper and lower limits of the input and output data of the neural network model used.

In this study, the values of the outputs are obtained from a model based on real boiler operation data. The outputs under study are predicted from four input datasets. Fig. 1 illustrates in detail the input and output variables of the neural network and its upper and lower limits.

**Fig. 2:**
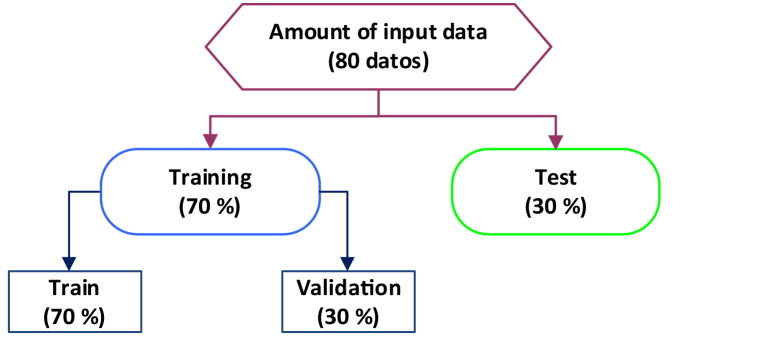
Graphic representation of dataset split for training, validation, and testing for the model.

The model input and output data were divided into two sets: training and testing at a proportion of 70 and 30%, respectively. The Train set is used to adjust the weights and parameters of the model, and the Test set is reserved for evaluating the final performance of the model after it has been fully trained. However, during the training process of the deep neural model, a subset called validation is created and represented from the total training dataset. It is considered a crucial procedure to control overfitting and select the appropriate hyperparameters that maximize model performance. Fig. 2 shows graphic representation of dataset split. The test data is not used in the training phase but only to evaluate the performance and examine the quality of the ML model [19].

In summary, including the “validation set” as an integral part of the training process in the deep neural model is essential to ensure that we obtain a well-fitted and generalizable model.

### 2.2 Deep neural networks for regression problems

Regression analysis is a subfield of supervised machine learning whose goal is establishing a method for the relationship between several features and a continuous target variable. In this sense, a deep neural network (DNN) has been designed as a machine learning technique to solve a regression problem from the defined independent and dependent variables.

**Fig. 3:**
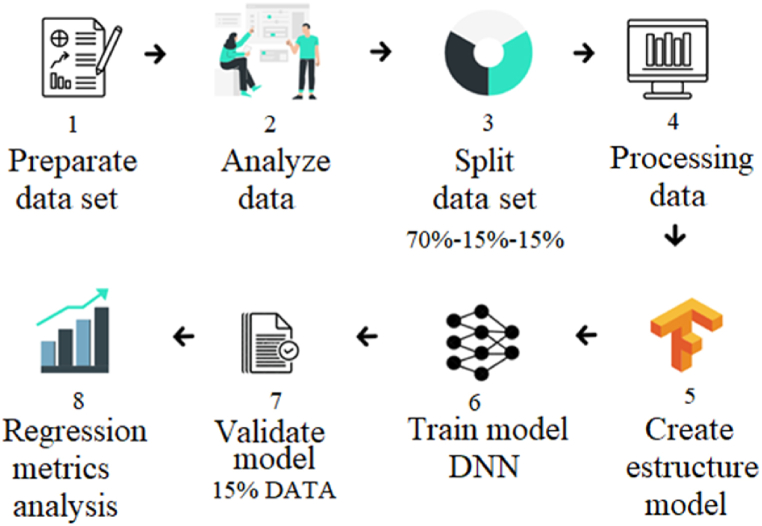
The sequence of steps for the implementation of the model.

The DNN model was built by establishing a sequence of steps, as shown in Fig. 3. First, the experimental dataset was organized into a historical document. Independent and dependent variables were then analyzed and selected, and continuous outputs were labeled. The data was then loaded, and the dataset was partitioned for pre-processing.

Z-score normalization was performed using *Sklearn's StandardScaler* class for the training and test sets. The z-normalization subtracts each data from its mean and divides it by the standard deviation of the data.

Subsequently, the structure of the deep regression model was created, and the deep neural model was adjusted. Finally, validation was performed on 15% of the data, and statistical regression metrics were analyzed for each set.

### 2.3 Structure of the deep neural model

**Fig. 4:**
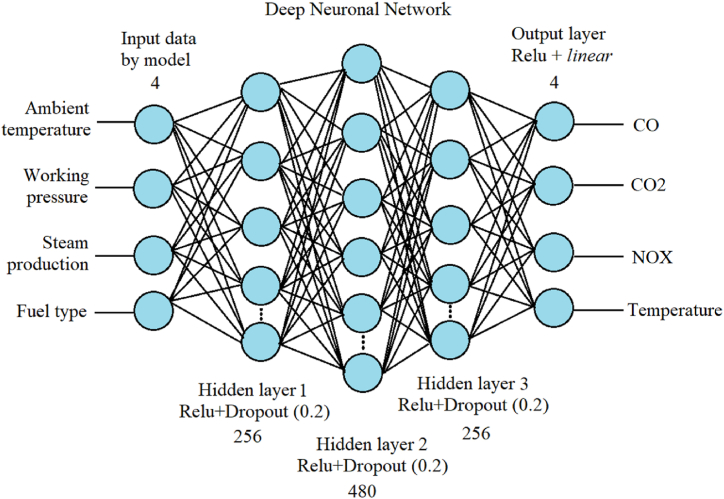
Structure of the deep neural model for the regression problem.

The neural model was built using the *TensorFlow* sequential class with a 4-layer architecture. The structure of the deep neuronal model illustrated in Fig. 4. The first dense layer consists of 256 hidden neurons with an input dimension size of 4, with an activation function *ReLu* and a normal-type initializer (*kernal_intializer*) to define the random weights.

The previous layer will relate to the second dense layer, with hidden units of 480, and a third layer consisting of 256 neurons, with activation function *ReLu* and *kernal_intializer* of the normal type. At each layer's end, the exclusion's regularization function is called *Dropout*. Subsequently, an output layer consisting of 4 neuron units, a normal *kernal_intializer,* and a *linear* type of activation function is defined.

Mean square logarithmic error (MSLE) was used as the loss function, an RMSprop type optimizer with a learning rate lr = 0.001. In addition, to evaluate the performance of the model during training and validation, the regression metrics Root Mean Square Error (RMSE), Mean Square Error (MSE), Mean Absolute Error (MAE), and the coefficient of determination (R^2^) were analyzed. The adjustment was made for a total of 1000 epochs.

The activation function is essential in the design of a neural network, even for solving complex nonlinear problems. Various activation functions have been applied to build DNNs, such as *Sigmoid*, *Tanh*, *Softplus*, and *ReLu*. Today, better performance is seen using the *ReLu* activation function [19].

Hinton introduced the so-called Dropout layer, which has proven to be very effective in reducing overfitting [20]. Dropout prevents the network from becoming too dependent on one or a small combination of neurons. As a result, it can force the network to be accurate even without specific information.

In [21], apply *Dropout* to fully connected layers, outperforming other methods studied without *Dropout*. In addition, it has been shown that there is a decrease in the error classification rate (ECR) when applying this layer in the network architecture.

### 2.4 Root mean square deep learning optimizer (RMS prop)

*RMS prop* is one of the most widely used optimizers in deep learning and is an advancement of the *AdaGrad* optimizer as it reduces the monotonically decreasing learning rate. The algorithm focuses mainly on speeding up the optimization process by reducing the number of function evaluations to reach the local minimum. In addition, the algorithm keeps the moving average of the squared gradients for each weight and divides the gradient by the square root of the mean square, equations (1), (2), (3)) [22,23],

(1) $$r_{k}=\left(1-\beta \right)∙{\left[\nabla_{x}f\;\left(x_{k}\right)\right]}^{2}+\beta r_{k-1}$$

(2) $$v_{k+1}=\frac{\eta }{\sqrt{r_{k}}}\nabla_{x}f\;\left(x_{k}\right)$$

(3) $$x_{k+1}=x_{k}-v_{k+1}$$

In simpler terms, updating this parameter is penalized if there is a parameter due to which the cost function fluctuates greatly. This algorithm has several advantages compared to previous versions of gradient descent algorithms. The algorithm converges quickly and requires fewer adjustments than gradient descent algorithms and their variants [24].

### 2.5 ML algorithms for regression problems

Tree-based methods have become one of the benchmarks in the predictive field due to the good results they generate in diverse problems. In this study, complex predictive models for regression, such as *Random Forest* and *Gradient Boosting,* were built to determine the model that achieves the most optimal balance between bias and variance. These models were implemented in Python through the *scikit-learn* library.

*Gradient Boosting* models are made up of a set of individual decision trees, trained sequentially so that each new tree tries to improve the errors of the previous trees. The prediction of a new observation is obtained by adding the predictions of all the individual trees that make up the model [25].

The Random Forest model is a combination of decision trees, so each tree depends on the values of a random vector tested independently and with the same distribution for each of these. It is considered one of the most accurate algorithms, widely used in problems of different complexity. In addition, it solves the problem of overfitting [25].

The Multiple Linear Regression (MLR) supervised learning algorithm is used when there is a linear relationship or dependence between the independent and dependent variables. This model was created in the study to determine if the predictor variables are linearly related to the response variables. In addition, this model is considered powerful due to its training speed and ease of understanding. For its implementation, the model proposed in Ref. [25] was used.

The hyperparameter settings, Table 2, for each model were designed to balance the complexity and generalizability of the models, avoiding over-fitting, and allowing the capture of relevant patterns in the data. This configuration improved model performance and adaptation to the specific task.

**Table 2:** Configuration of the hyperparameters for each of the models.

Models	Hyperparameters
Random Forest Regressor	n_estimators = 100 max_depth = min_samples_split = 3 min_samples_leaf = 1 max_features = “auto random_state = 42 n_jobs = −1
Gradient Boosting Regressor	n_estimators = 100 learning_rate = 0.1 max_depth = 3 min_samples_split = 2 min_samples_leaf = 1 max_features = None

### 2.6 Metrics for regression analysis

For the regression task, typical accuracy metrics studied were mean square error (MSE), root means square error (RMSE), mean absolute error (MAE), and coefficient of determination R^2^. These metrics measure the distance between the predicted numerical target and the actual numerical response (actual data). The definitions of these statistical metrics are described below.

The MSE is the simplest and most common metric for regression evaluation, but it is also probably the least useful. It is defined by equation (4).

(4) $$MSE=\frac{1}{N}\sum_{i=1}^{N}{\;\left(y_{i}-{\hat{y}}_{i}\right)}^{2}$$

where *yᵢ* is the actual expected result, and *ŷᵢ* is the model prediction.

MSE measures the mean squared error of our predictions. For each point, compute the squared difference between the predictions and the target and then average those values [25]. The higher this value, the worse the model. It is never negative since we are squaring the individual forecast errors before adding them together, but it would be zero for a perfect model.

RMSE is just the square root of MSE. The square root is introduced to make the scale of the errors equal to the scale of the targets. The root mean square error (RMSE) is the standard deviation of the residuals (prediction errors). Residuals are a measure of how far apart the data points are from the regression line; the RMSE is a measure of the dispersion of these residuals. The root mean square error is commonly used in regression analysis to verify experimental results, equation (5), [[26], [27], [28]].

(5) $$RMSE=\sqrt{\frac{1}{n}\sum_{i=1}^{n}{\;\left({\hat{y}}_{i}-y_{i}\right)}^{2}}$$

where *ŷ*ᵢ represents the predicted values and *y* the actual values.

The mean absolute error (MAE) loss is a suitable loss function in this case, as it is more robust against outliers. Therefore, it is calculated as the mean of the absolute difference between the actual and predicted values, equation (6). The model can be updated to use the ‘mean_absolute_error’ loss function and keep the same settings for the output layer [29,30].

(6) $$MAE=\frac{1}{n}\sum_{i=1}^{n}\left|y_{i}-{\hat{y}}_{i}\right|$$

The R-squared is the statistical measure determining how close the data is to the fitted regression line. It is also known as the coefficient of determination or multiple coefficients of determination if it is multiple regression, equation (7), [29].

(7) $$R^{2}=1-\frac{\sum_{i=1}^{n}{\left(y_{i}-{\hat{y}}_{i}\right)}^{2}}{\sum_{i=1}^{n}{\left(y_{i}-{\hat{y}}_{i}\right)}^{2}}$$

The coefficient of determination, or R^2^ (sometimes read as R-two), is another measure we can use to evaluate a model and is closely related to the MSE but has the advantage of being scale-free, no matter if the output values are very large or very small, the R^2^ will always be between -∞ and 1. When R^2^ is negative, the model is worse than predicting the mean.

The greater the variance the regression model explains, the closer the data points are to the fitted regression line. In theory, if a model could explain 100% of the variance, the fitted values would always equal the observed values, and therefore, all data points would lie on the fitted regression line.

## 3 Results and discussion

**Fig. 5:**
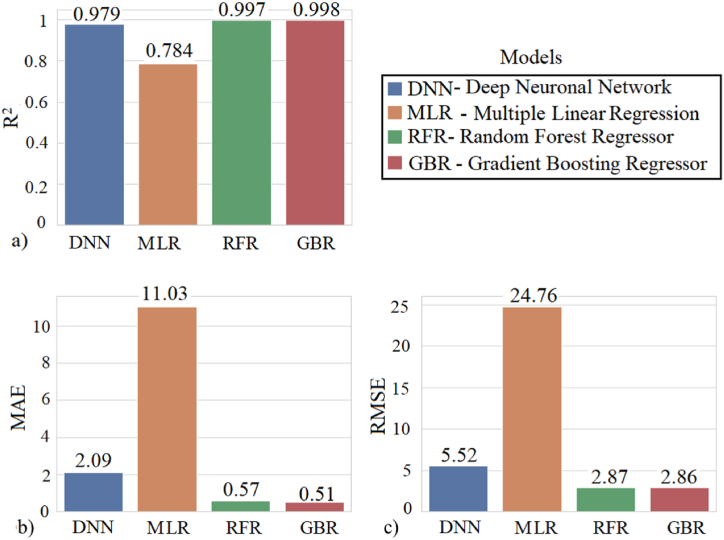
Graphic representation of the metrics **(**a) R^2^: R-squared, (b) MAE: mean absolute error and (c) RMSE: root mean square error obtained for each model.

The preparation of the dataset, the design of the ML models, and the techniques applied to reduce overfitting contributed to the prediction of the study output variables. The objective of the analysis was to train and evaluate the regression metrics once the models were fitted. Fig. 5 shows bar charts with the results of the statistical metrics calculated for the test dataset.

The Gradient Boosting Regressor, Random Forest Regressor and the DNN models outperform the Multiple Linear Regression model by a wide margin. The bar chart illustrated in Fig. 5 (a) shows that the RGB model had a high coefficient of determination with respect to the other models. This result corresponds to the mean absolute error and the root mean square error represented in the graphs in Fig. 5(b–c) respectively. Next, each of the results of these models will be analyzed.

Table 3 discusses the results obtained for the training set and the validation of the deep neural model. In addition, regression metrics were calculated, which made it possible to study the behavior of the data against the designed network structure.

**Table 3:** Results of the regression metrics to evaluate the deep neural model.

Metrics	Training Set	Validation Set
RMSE	6.95	6.78
MSE	48.28	46.05
MAE	2.66	2.46
R^2^	0.96	0.94

**Fig. 6:**
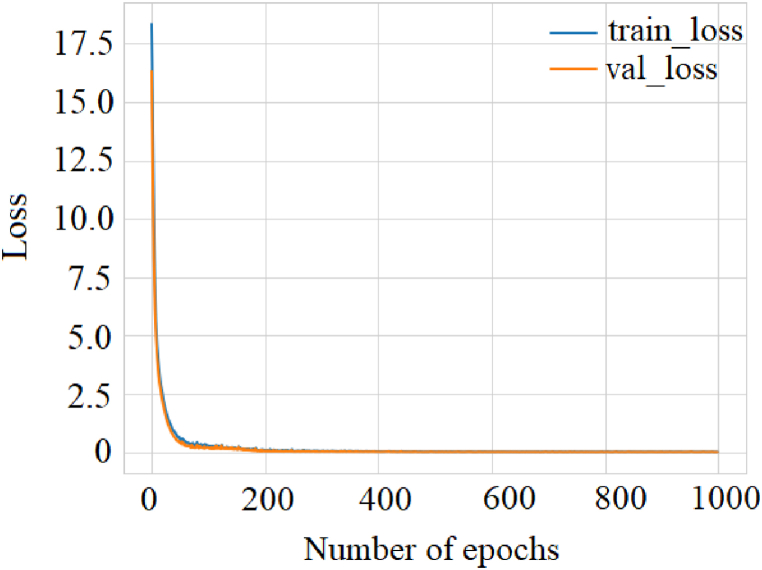
Result of the loss function of the model through the MSLE.

The regression metrics were graphically described to understand the results better. Considering the dispersion of the data in this study, the loss of the model was analyzed by calculating the MSLE, Fig. 6. As the epochs increased, the MSLE algorithm converged.

**Fig. 7:**
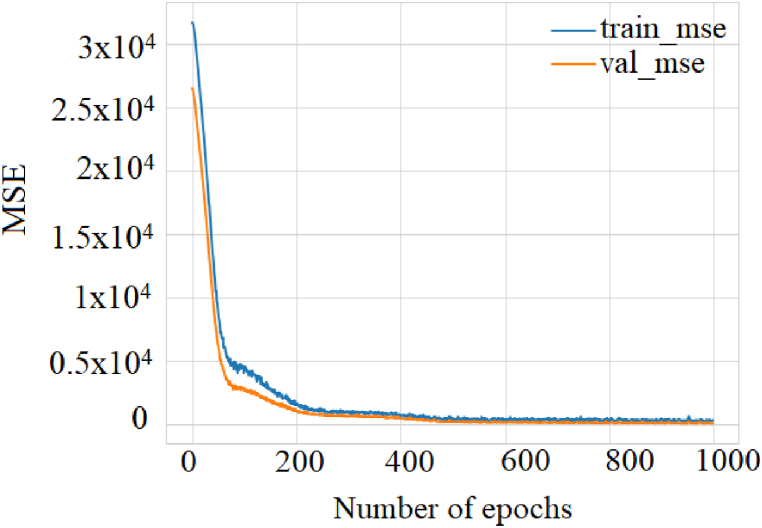
MSE metric to assess model accuracy.

On the other hand, the RMSE and MSE metrics were evaluated, and the latter calculated the average of the squared difference between the predictions and the target. The RMSE is just the square root of the MSE. The square root is introduced to make the scale of the errors equal to the scale of the targets. Fig. 7 shows the results obtained for the MSE metric.

**Fig. 8:**
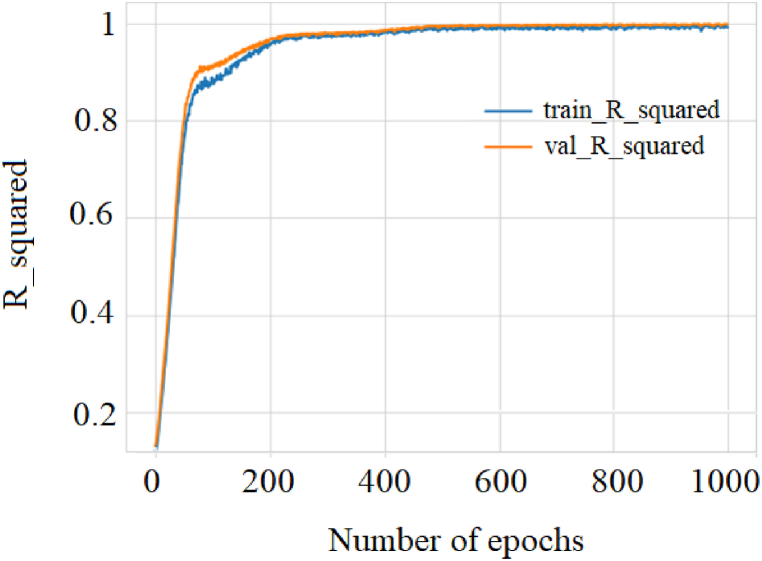
Evaluation of the regression metric R^2^ for training and validation.

As can be seen, as the stages increased, a lower MSE value was reached and, consequently, the RMSE. This result implies a higher precision of the regression model. RMSE and R^2^ quantify how well a deep regression model fits the dataset. Fig. 8 shows how the predictor variables can explain the variation of the response variable for the training and validation set.

**Fig. 9:**
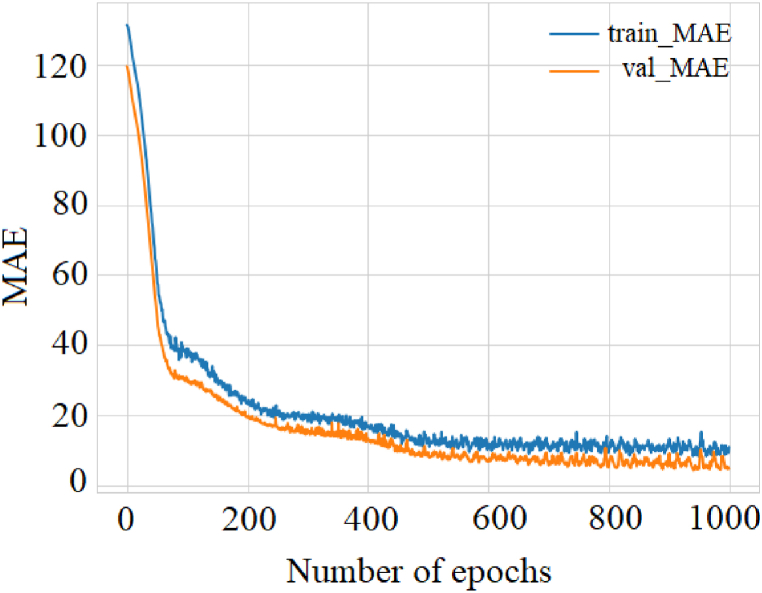
Evaluation of the performance of the neural model through the MAE metric.

Notably, the R^2^ and the MSE are closely related measures; the correlation coefficient increases as the MSE values decrease. Since the study data contains outliers, it was also decided to evaluate the performance of the regression model through the MAE measure. Fig. 9 shows the frequency with which the predictions measure the target. The mean absolute error between the expected and predicted values decreased as the epochs increased. However, note the difference in the adjustment for the training and validation datasets, behavior associated with the MAE calculated against the test data with a value of 2.09, which will later be compared with the rest of the data models under study.

When selecting the appropriate model for the prediction of the emission of CO_2_, CO, NO_X,_ and the outlet temperature of the exhaust gases, it was decided to study other regression models. Therefore, the algorithms for Multiple Linear Regression, Gradient Boosting Regressor, and Random Forest Regressor were used, and the results were compared against the deep neural model, Table 4.

**Table 4:** Comparison of the performance of the different models.

Model	RMSE	MSE	MAE
DNN	5.52	30.46	2.09
Multiple Linear Regression (MLR)	24.76	612.85	11.03
Random Forest Regressor (RFR)	2.87	8.28	0.57
Gradient Boosting Regressor (GBR)	2.86	8.18	0.51

It is important to note that the DNN regression model explains 97.90% of the variance and a MAE of 2.09 compared to the test set. Compared to the Multiple Linear Regression model, the DNN model fits the real values better, concluding that the deep neural models greatly outperform the Multiple Linear Regression models. This means that the higher the variance explained by the regression model and for a smaller MAE value, the closer the data will be to the fitted regression line.

**Fig. 10:**
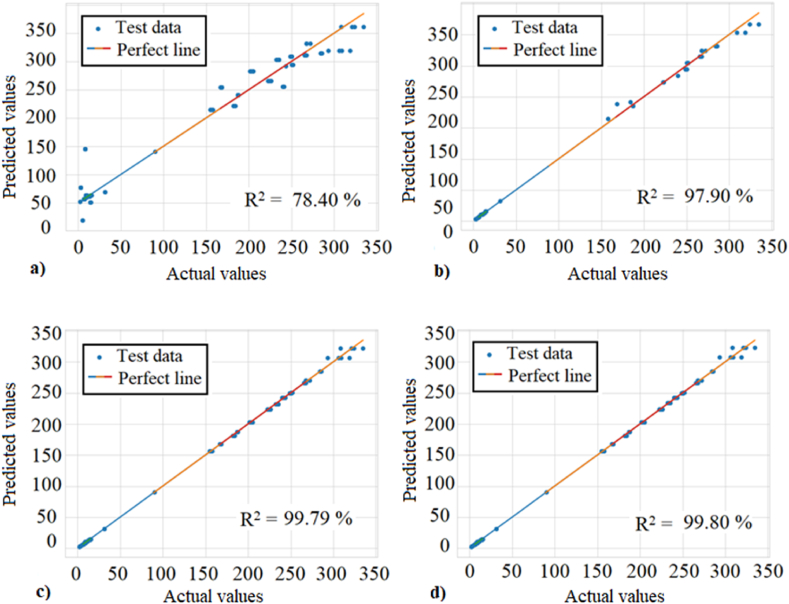
Comparison of scatter plots of actual values versus predicted values in the test dataset for each model (a) MLR: Multiple Linear Regression, (b) DNN: Deep Neural Network, (c) RFR: Random Forest Regressor, and (d) GBR: Gradient Boosting Regressor. The blue points represent the test dataset, and the multicolored line represents the fitted regression line.

Fig. 10 shows scatter plots of the different regression models to compare the results of the models that best fits the fitted regression line in the test phase. Furthermore, in this comparison shows the calculated R-squared value in order to assess the performance of the models to predict the actual values.

In the scatter plot for the MLR model, as shown in Fig. 10 (a), the observations or actual values are further away from the fitted regression line and an R^2^ explaining 78.40% of the variance, indicating that the model is not explaining the variability in the test data well. However, for the DNN, RFR and GBR models in Fig. 10(b–d) reveal that the observations are closer to the fitted regression line and explains 97.90, 99.79 and 99.80 % of the variance respectively. It is confirmed that the GBR model represented in Fig. 10 (d) was the best fitting model with a variance of 99.80%.

The superiority of GBR compared to RFR in this specific exhaust emission prediction problem is due to its ability to fit non-linear relationships. This can be important in exhaust emissions problems where multiple environmental and operational factors interact in complex ways [31].

GBR is an algorithm that trains decision trees sequentially, where each tree attempts to correct the errors of the previous tree, allowing it to improve its predictions gradually. The choice of the GBR model due to its sequential structure tends to outperform RFR in predictive performance, especially on moderate to big datasets [32]. This is due to its ability to build a more flexible and data-adaptive model. On the other hand, although RFR is equally accurate [33], it may require a more significant number of trees to achieve similar performance in complicated situations. Both models are resilient in robustness to overfitting, but GBR, due to its sequential approach, tends to be less prone to overfitting than RFR. However, regarding speed, RFR tends to outperform GBR because each decision tree in a random forest is trained independently, facilitating parallelization.

GBR gives more weight to the examples mispredicted in the previous trees, which helps the model focus on the most difficult cases to predict. This capability can be crucial in a gas emissions problem where relationships can be variable and dependent on multiple factors.

Therefore, the Gradient Boosting Regressor machine learning algorithm was the appropriate model to predict the emission of CO_2_, CO, NOx, and the exhaust gas outlet temperature in this study.

CO, CO_2_, NO_X_, and temperature gas formation in combustion is a complex process that occurs when a fuel is burned in the presence of oxygen. The amount and composition of these gases depend on the type of fuel used and the combustion conditions, such as temperature, pressure, and the amount of oxygen present. Carbon dioxide (CO_2_) is formed when carbon in the fuel combines with oxygen in the air. Carbon monoxide (CO) is produced when the fuel does not burn completely, and the oxygen does not combine with all the carbon present. Nitrogen oxides (NO_X_) are formed when nitrogen in the air reacts with oxygen at high temperatures. Flue gas temperature is directly related to combustion efficiency [34].

This aspect has been studied extensively and is known and discussed in different bibliographies specialized in combustion, which is why this subject is not addressed in the article presented.

**Fig. 11:**
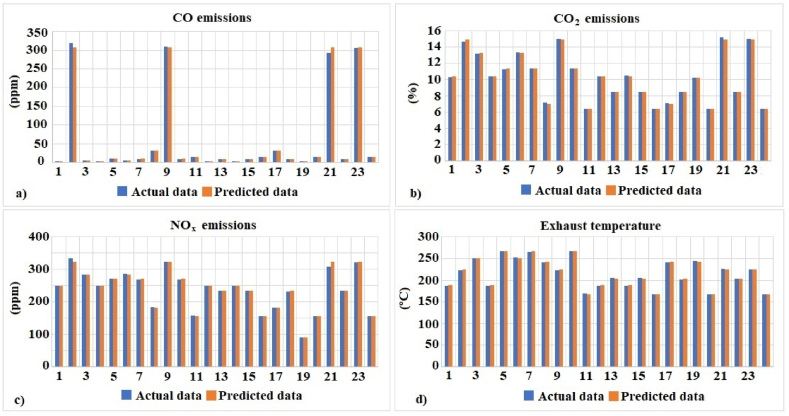
Graphic illustration of predicted and actual values for each output target: (a) CO emissions, (b) CO_2_ emissions, (c) NOx emissions and (d) Exhaust temperature.

To analyze in depth the results obtained in the prediction of the output variables, as well as to make a comparison of these results with the experimental ones, both variables were plotted. Fig. 11 shows the high correspondence between the experimental data and those predicted by the proposed model.

Fig. 11(a–d) shows that the predicted values (in blue) closely follow the actual values (in orange). In Fig. 11(a), the discrepancy between the two datasets is minimal. In Fig. 11 (b), we again observe a similarity between the predicted and actual values. This indicates that our model is reliable in predicting CO_2_ emissions. Fig. 11 (c) shows a similar trend, with the predicted values closely overlapping the actual values. The difference is minimal. Finally, in Fig. 11 (d), we see an agreement between the predicted and actual values of the exhaust temperature. This suggests that the model is accurate in predicting the variables.

When analyzing the results presented in the graphs, several conclusions can be drawn: the regression model demonstrates the ability to predict CO, CO_2_, and NOx emissions and the exhaust gas outlet temperature. The differences between predicted and experimental values are minimal for all parameters analyzed. No consistent trend of under- or over-estimation is observed for any of the variables. Differences between predicted values and experimental values can be attributed to several reasons: small variations in operating conditions or input data during testing could affect the results.

The high quality of the predictions, supported by an R^2^ of 99.80% and an error of 0.51, validates the effectiveness of the GBR regression model. This suggests that the model is a reliable tool for estimating CO, CO_2_, NOx emissions, and exhaust gas outlet temperature in the study context. In summary, the emissions and temperature analyses demonstrate that the regression model is highly accurate and reliable. The results obtained reinforce the quality of the predictions.

Therefore, it was concluded that the Gradient Boosting Regressor machine learning algorithm is the appropriate model to predict the emission of CO, CO_2_, NOx, and the exhaust gas outlet temperature in this study.

## 4 Conclusions

In this research, the modeling of gas emissions from real data collected from 20 industrial firetube boilers in Santiago de Cuba using a Testo 350 gas analyzer was addressed. Environmental and operational variables, such as ambient temperature, working pressure, steam production, and fuel type related to CO, CO_2_, NOx emissions, and gas outlet temperature, were considered.

The selection of variables was based on their relevance from environmental and combustion efficiency perspectives. This is crucial for pollution control and efficiency improvement in industrial operations.

Several machine learning algorithms, including Linear Regression, Gradient Boosting, Random Forest, and a deep neural model (DNN), were explored to identify the most appropriate model for predicting emissions and gas temperatures.

The results revealed that the Gradient Boosting Regressor-based model showed the best prediction performance on the test data, outperforming all other models studied. A low mean absolute error of 0.51 and a high coefficient of determination of 99.80% were achieved. This demonstrates the effectiveness of the machine learning approach in predicting emissions and temperatures in this context.

It was observed that the DNN model outperformed traditional regression models, highlighting its ability to deal with complex data and improve the accuracy of predictions.

Implementing these artificial intelligence methods, especially the Gradient Boosting Regressor model, offers the possibility to reduce the frequency of gas analysis in industrial boilers. This represents a saving of resources and time, as only the input variables need to be updated to monitor the parameters of interest.

This research provides an effective and efficient method for predicting gas emissions in industrial boilers with significant environmental and operational management applications.

## Funding

None.

## Data availability statement

Data included in article/supplementary material/referenced in article. Data will be made available on request.

## Additional information

No additional information is available for this paper.

## CRediT authorship contribution statement

**Bárbara D. Ross-Veitía:** Supervision, Software, Resources, Project administration. **Dayana Palma-Ramírez:** Investigation, Formal analysis, Data curation, Conceptualization. **Ramón Arias-Gilart:** Writing – original draft. **Rebeca E. Conde-García:** Validation, Resources, Project administration, Funding acquisition, Formal analysis. **Alejandro Espinel-Hernández:** Validation, Supervision, Project administration, Investigation, Formal analysis, Data curation, Conceptualization. **José R. Nuñez-Alvarez:** Writing – review & editing, Writing – original draft, Validation, Supervision, Software, Project administration, Investigation, Funding acquisition. **Hernan Hernández-Herrera:** Visualization, Validation, Supervision, Project administration, Funding acquisition. **Yolanda E. Llosas-Albuerne:** Writing – review & editing, Validation, Conceptualization.

## Declaration of competing interest

The authors declare that they have no known competing financial interests or personal relationships that could have appeared to influence the work reported in this paper.
